# Human DDX17 Unwinds Rift Valley Fever Virus Non-Coding RNAs

**DOI:** 10.3390/ijms22010054

**Published:** 2020-12-23

**Authors:** Corey R. Nelson, Tyler Mrozowich, Sean M. Park, Simmone D’souza, Amy Henrickson, Justin R. J. Vigar, Hans-Joachim Wieden, Raymond J. Owens, Borries Demeler, Trushar R. Patel

**Affiliations:** 1 Department of Chemistry and Biochemistry, Alberta RNA Research and Training Institute, University of Lethbridge, 4401 University Drive, Lethbridge, AB T1K 3M4, Canada; corey.nelson@uleth.ca (C.R.N.); tyler.mrozowich@uleth.ca (T.M.); sean.park@uleth.ca (S.M.P.); amy.henrickson@uleth.ca (A.H.); Justin.vigar@mail.utoronto.ca (J.R.J.V.); hj.wieden@uleth.ca (H.-J.W.); demeler@gmail.com (B.D.); 2 Department of Microbiology, Immunology and Infectious Disease, Cumming School of Medicine, University of Calgary, Calgary, AB T2N 1N4, Canada; simmone.dsouza@ucalgary.ca; 3 Research Complex at Harwell, R92 Rutherford Appleton Laboratories, Harwell, Oxford OX1 0QX, UK; ray@strubi.ox.ac.uk; 4 Department of Chemistry and Biochemistry, University of Montana, Missoula, MT 59812, USA; 5 NorthWest Biophysics Consortium, University of Lethbridge, University of Lethbridge, 4401 University Drive, Lethbridge, AB T1K 3M4, Canada; 6 Li Ka Shing Institute of Virology and Discovery Lab, University of Alberta, Edmonton, AB T6G 2E1, Canada

**Keywords:** Rift Valley fever virus RNA, helicase DDX17, host–viral interactions, analytical ultracentrifuge, microscale thermophoresis, fluorescent labeling, small angle X-ray scattering, helicase assay

## Abstract

Rift Valley fever virus (RVFV) is a mosquito-transmitted virus from the *Bunyaviridae* family that causes high rates of mortality and morbidity in humans and ruminant animals. Previous studies indicated that DEAD-box helicase 17 (DDX17) restricts RVFV replication by recognizing two primary non-coding RNAs in the S-segment of the genome: the intergenic region (IGR) and 5′ non-coding region (NCR). However, we lack molecular insights into the direct binding of DDX17 with RVFV non-coding RNAs and information on the unwinding of both non-coding RNAs by DDX17. Therefore, we performed an extensive biophysical analysis of the DDX17 helicase domain (DDX17_135–555_) and RVFV non-coding RNAs, IGR and 5’ NCR. The homogeneity studies using analytical ultracentrifugation indicated that DDX17_135–555_, IGR, and 5’ NCR are pure. Next, we performed small-angle X-ray scattering (SAXS) experiments, which suggested that DDX17 and both RNAs are homogenous as well. SAXS analysis also demonstrated that DDX17 is globular to an extent, whereas the RNAs adopt an extended conformation in solution. Subsequently, microscale thermophoresis (MST) experiments were performed to investigate the direct binding of DDX17 to the non-coding RNAs. The MST experiments demonstrated that DDX17 binds with the IGR and 5’ NCR with a dissociation constant of 5.77 ± 0.15 µM and 9.85 ± 0.11 µM, respectively. As DDX17_135–555_ is an RNA helicase, we next determined if it could unwind IGR and NCR. We developed a helicase assay using MST and fluorescently-labeled oligos, which suggested DDX17_135–555_ can unwind both RNAs. Overall, our study provides direct evidence of DDX17_135–555_ interacting with and unwinding RVFV non-coding regions.

## 1. Introduction

Rift Valley fever virus (RVFV) is part of the *Bunyaviridae* family and the genus *Phlebovirus*. The virus was first identified in the early 1930s during a large outbreak on a sheep farm in the Rift Valley of Kenya [1]. Since then, the virus transmission has been reported in several countries located within Sub-Saharan Africa and the Arabian Peninsula due to infected livestock trade. Transmission of this virus is through competent mosquito vector hosts, Aedes and Culex, to animals and humans. Infection is currently untreatable and primarily affects domesticated animals such as camels, goats, sheep, and cattle, but can also affect humans [2]. Infections in animals mainly occur through mosquito bites, and terminal human hosts can be infected by infected mosquitos and direct contact with infected ruminant blood and bodily fluids. Symptoms of RVFV infection in humans can include acute febrile illness followed by hemorrhagic fever, encephalitis, or ocular disease [2]. These mosquito vectors can also transmit a variety of flaviviral diseases such as Zika, yellow fever, and chikungunya. Additionally, its presence on every continent except for Antarctica makes this virus a severe threat to global health and food security [3]. There is currently an inactivated vaccine (MP-12) that has been shown to confer long-term immunity in humans with a single dose. This vaccine is also effective in animals; however, it is not currently commercially available [4].

According to the World Health Organization (WHO), RVFV infection is one of the top eight emerging diseases likely to cause significant epidemics that currently have no medical countermeasures [5]. RVFV is maintained in the environment via vertical transmission from the mosquito vector to offspring during periods of high rainfall, which amplifies mosquito breeding [6]. Infectious outbreaks occur after long intervals of dormancy, between 5–15 years, but can cause detrimental economic losses due to livestock infection, which causes mortality rates of 10–30% and >90% abortion rates. Moreover, these epizootic outbreaks have resulted in a total death toll of over 100,000 sheep, over half a million livestock abortions, and more than 2300 human deaths [1,7].

RVFV is an enveloped virus that contains a linear, tripartite, ssRNA ambisense genome [8]. The total tripartite genome size for RVFV is 10.4 kb and encompasses three different viral RNA components which are replicated in the host cell cytoplasm: L (large, 6.4 kb) and M (medium, 2.3 kb), which are both negative sense, and S (small, 1.7 kb) which is ambisense [9]. The L-segment encodes the RNA-dependent RNA polymerase, while the M-segment encodes two envelope glycoproteins, Gn and Gc, and two accessory proteins. The S-segment (S2) ambisense RNA encodes a positive-sense RNA template of the non-structural protein (NS) and a negative-sense RNA for the viral nucleoprotein (N) [10,11]. The S-segment contains two notable non-coding regions; the 5′ non-coding region (NCR), responsible for transcription and translation initiation of the NS segment, and the intergenic region (IGR), responsible for transcription termination on both the NSs and N mRNA [12]. These two regions have been demonstrated to form hairpin-like structures, which are recognized by host cellular machinery. The human protein, DDX17, which is a DEAD-box helicase, has been shown to interact with the 5′ non-coding S-segment (RVFV NCR) and the non-coding sequence between N and NSs (RVFV IGR) [11]. The knockdown of DDX17, but not its paralog DDX5, led to unrestricted viral replication of RVFV in U2OS cells [11].

DEAD-box helicases have been described as ATP-dependent chaperones that reconfigure RNA by disrupting secondary and tertiary RNA–RNA or RNA–protein interactions [13,14]. The DDX17 helicase has roles in transcription, splicing, mRNA decay, rRNA biogenesis, and miRNA processing as well as antiviral defense [15,16]. The target motifs on the viral RNA S-segment are two hairpin structures, which are unique to the *Bunyaviridae* family [10]. CLIP-seq data indicate that DDX17 interacts with both the IGR and 5’ NCR of the RVFV S-segment. We wanted to examine these interactions in vitro to determine if DDX17 is capable of functioning independently or whether it requires additional binding partners, as described previously [17]. Additionally, we would like to investigate the structural differences between the non-coding RNAs (ncRNAs) and how this could affect their interactions with DDX17.

Therefore, by utilizing multiple biophysical techniques, we have characterized the interaction between RVFV non-coding RNA (ncRNA) and DDX17_135–555_ to determine that not only does DDX17_135–555_ directly interact with RVFV ncRNA, it also unwinds the ncRNA in the presence of ATP. This work supports previous observations of direct anti-viral effects of DDX17 [11] while providing a new, easy approach to investigate the helicase activity of a protein.

## 2. Results

### 2.1. Purification of DDX17_135–555_, RVFV S-segment IGR, and 5′NCR

**Figure 1:**
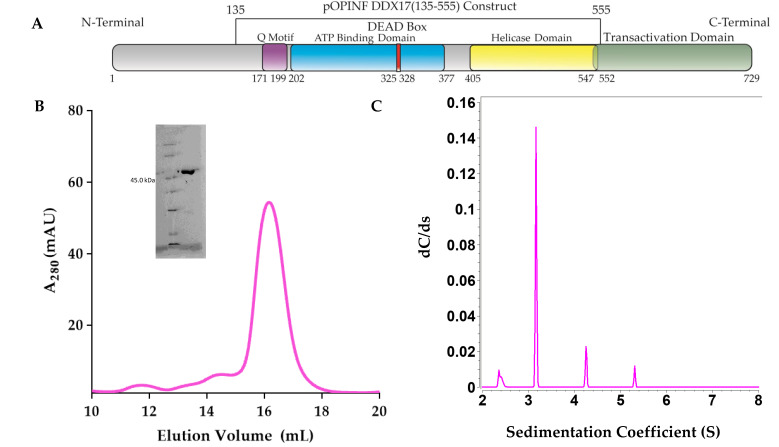
(**A**) Schematic representation of DDX17 highlighting individual domains. DDX17_135–555_, which contains the Q motif, ATP binding domain, DEAD-box, and the helicase domain, was used in downstream studies. (**B**) The chromatogram from the size exclusion purification (Superdex 200 Increase gl 10/300) of DDX17_135–555_, suggesting that DDX17_(135–555)_ can be purified to ~68% homogeneity (~16 mL). The y-axis represents absorbance at 260 nm while the x-axis represents elution volume. We collected peak fractions from 15.5 to 16.5 mL for subsequent analysis. The inset to Figure 1B represents the SDS-PAGE analysis of DDX17_135–555_ (48.45 kDa) following size exclusion chromatography. (**C**) Sedimentation coefficient distribution of DDX17_135–555_ obtained from analytical ultracentrifugation sedimentation velocity (SV-AUC) experiment. The peak at ~3.16S represents monodispersed DDX17_135–555_. Sedimentation coefficient values are corrected to standard solvent conditions (20 °C in water).

DDX17_135–555_ was overexpressed in *Escherichia coli* (Lemo21) and purified using affinity and size exclusion chromatography (SEC), as detailed in the Materials and Methods section. Figure 1A shows the schematics of full-length DDX17 and the truncated DDX17_135–555_ that was used in this study, as we were unable to express sufficient amounts of full-length DDX17. As presented in Figure 1B, the peak fractions (15 mL to 17 mL), devoid of any contamination or aggregation, were collected, followed by the purity check using SDS-PAGE. As presented in the inset to Figure 1B, the final preparation does not contain any degraded material, and it corresponds to the correct molecular weight of ~50 kDa. To further study the homogeneity of DDX17_135–555_ in solution, the SEC-purified preparation that presented a single band in SDS-PAGE was used to perform an analytical ultracentrifugation sedimentation velocity experiment (SV-AUC). The SV-AUC results suggest that DDX17_135–555_ is mainly homogenous with a sedimentation coefficient of 3.16 S (Figure 1C) and a diffusion coefficient of 5.22 × 10^−7^ cm^2^/s (Table 1).

**Figure 2:**
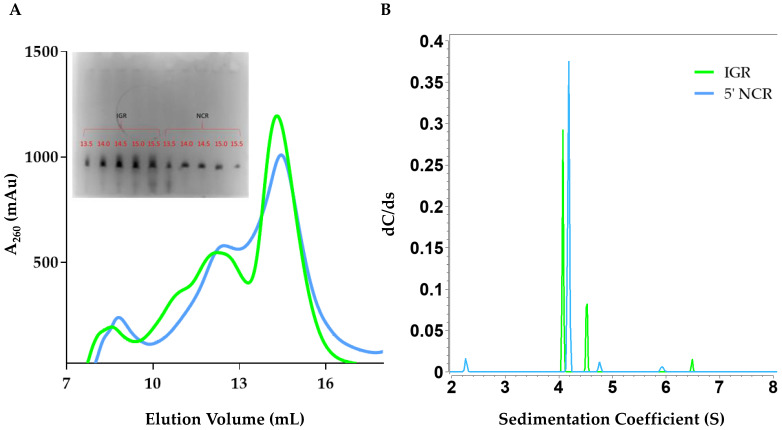
Purification and hydrodynamic characterization of in vitro transcribed Rift Valley fever virus RNA. (**A**) Size exclusion chromatogram of the elution profile of both Rift Valley fever virus (RVFV) 5’ intergenic region (IGR) and RVFV 5’ non-coding region (NCR). The y-axis represents absorbance at 260 nm while the x-axis represents elution volume. An inset to Figure 2A represents the urea-PAGE (7.5%) analysis of RVFV IGR and 5’ NCR after size exclusion chromatography. Each well represents 10 µL of a 500 µL elution fraction from size exclusion chromatography. The gel was run for 25 min, at 300 V in 0.5× TBE (Tris-Borate-EDTA) running buffer and was visualized using Sybr Safe dye. (**B**) Sedimentation coefficient distribution profiles for RVFV 5’ IGR (green) and RVFV 5’ NCR (blue) from SV-AUC. The primary SV peaks for each RNA are 4.07 S and 4.18 S for IGR and 5’ NCR, respectively, and represent the monomeric form. Sedimentation coefficient values were corrected to standard conditions (20 °C in water).

The in vitro transcribed RVFV IGR and 5’ NCR RNAs were purified using SEC, similar to DDX17_135–555_ (Figure 2A). The IGR eluted at approximately ~14 mL, while the 5’ NCR eluted at ~14.5 mL. Peak fractions were collected and analyzed by urea-PAGE, which displayed a single band (Figure 2A inset). Next, we utilized SV-AUC to determine the purity of SEC-purified RVFV ncRNA. Our SV-AUC analysis suggested that monomeric IGR and 5’ NCR have sedimentation coefficients of 4.07 S and 4.18 S, respectively. The SV-AUC analysis also yielded diffusion coefficients of 7.62 × 10^−7^ cm^2^/s and 6.58 × 10^−7^ cm^2^/s, respectively. Overall, both ncRNAs appear to be relatively pure (Figure 2B).

### 2.2. Solution Conformation of DDX17_135–555_, RVFV S-Segment IGR, and 5′NCR

**Figure 3:**
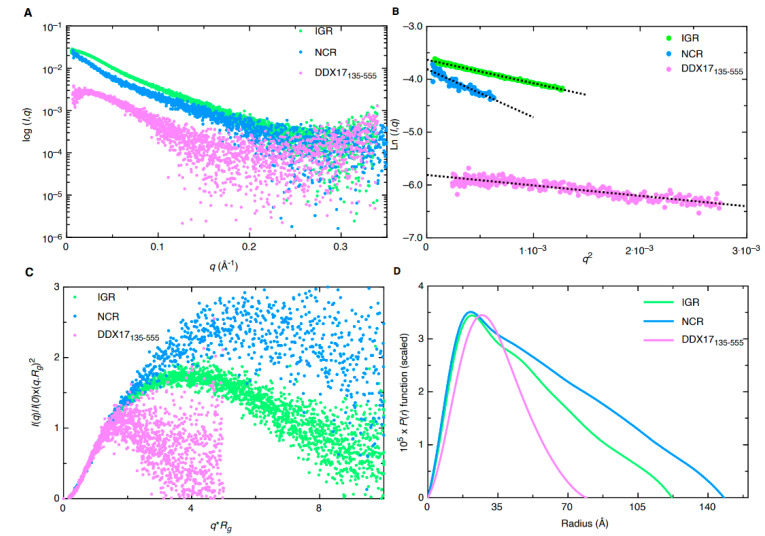
Small-angle X-ray scattering (SAXS) characterization of RVFV RNA (IGR and NCR) and DDX17_135–555_. (**A**) Merged scattering data of RVFV RNA and DDX17_135–555_ showing scattering intensity (log I(q)) vs. scattering angle (q = 4πsinθ/λ). (**B**) Guinier plots allowing for the determination of R_g_ from low-angle region data and representing the homogeneity of samples. (**C**) Dimensionless Kratky plots (I(q)/I(0)*(q*R_g_)^2^ vs. q*R_g_) of RVFV RNA and DDX17_135–555_, demonstrating extended structures for RVFV RNA and a more compact structure for DDX17_135–555_. (**D**) Pair distance distribution (P(r)) plots for RVFV RNA and DDX17_135–555_ which allow for the determination of R_g_ from the entire SAXS dataset, and maximal particle dimension (D_max_).

SAXS analysis allows for low-resolution structural determination of biomolecules in solution. The instrumentation provided at the B21 Beamline (Diamond Light Source, UK) allows for the employment of HPLC connected in-line to SAXS detection to maintain confidence in the monodispersity of samples, keeping them free of aggregates and degradation [18,19,20]. SEC-SAXS data for the merged datasets are presented in Figure 3A. The merged data were further processed using Guinier analysis (plot of (l(q)) vs. (q^2^)) to detect the purity and for the determination of the R_g_ (average root mean squared radius from the center of mass for the biomolecule) from the low-q region [21]. Figure 3B represents the Guinier plots for IGR, 5’ NCR, and DDX17_135–555_, whereas the linearity of the low-q region indicates that all three biomolecules were monodisperse. R_g_ values of 36.42 ± 0.10, 50.44 ± 0.88, and 24.78 ± 0.36 for IGR, 5’ NCR, and DDX17_135–555_, respectively, were obtained from Guinier analysis (see Table 1). After we confirmed monodispersity from Guinier analysis, we further processed the SAXS scattering data from Figure 3A to obtain dimensionless Kratky plots [19,22] which allowed for analysis of the foldedness of the biomolecules (Figure 3C). In general, globular biomolecules in solution show a well-defined maximum value of 1.1 at q*R_g_ = 1.73 [23]. The dimensionless Kratky plots for the two ncRNAs suggested that both are well folded and extended in solution, whereas DDX17_135–555_ is relatively more compact.

Next, indirect Fourier transformations on each dataset were performed to convert the reciprocal-space information of data presented in Figure 3A to real-space electron pair distance distribution functions (P(r)) plots, which are presented in Figure 3D using the GNOM [24] program. Using the P(r) plots, the R_g_ was obtained along with the D_max_ (maximal particle dimension) for all three biomolecules. Importantly, compared to Guinier analysis, which provides R_g_ from the low-q region, the P(r) analysis utilizes a larger range of the dataset which adds to the reliable determination of the R_g_ and D_max_. Table 1 contains all values calculated from the P(r) analysis; we obtained a D_max_ of ~120 Å, 145 Å, and 80 Å for IGR, 5’ NCR, and DDX17_135–555_, respectively. Additionally, we obtained P(r) R_g_ values of 38.00 ± 0.08, 46.66 ± 0.34, and 25.46 ± 0.27 Å for IGR, 5’ NCR, and DDX17_135–555_, respectively. These values correlate very well to those obtained from prior Guinier analysis, indicating these data are suitable to proceed with low-resolution structure determination. The P(r) plot is also indicative of a biomolecules’ relative solution conformation; a more globular-shaped biomolecule will adopt a bell-shaped P(r) distribution with a maximum at D_max_/2 [25], and a more extended molecule will adopt a bell-shaped curve with an extended tail, suggesting an elongated structure [20]. The P(r) plot for DDX17_135–555_ adopts a typical bell-shaped curve, which suggests that this protein is more globular relative to the ncRNAs (Figure 3D).

**Figure 4:**
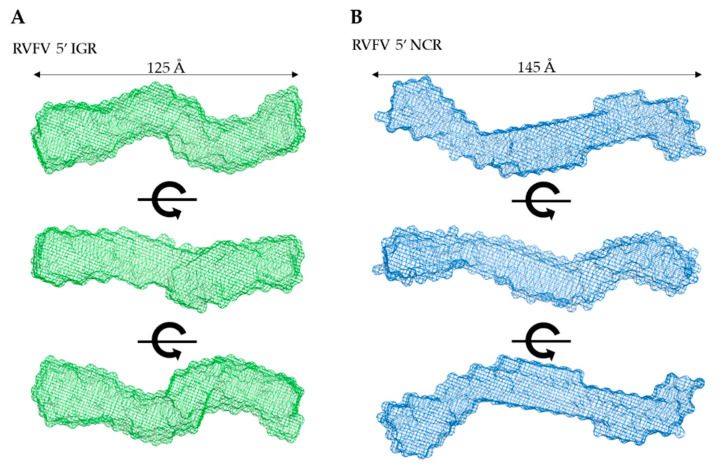
Low-resolution structure determination via SAXS for RVFV 5’ NCR and RVFV 5’ IGR, indicating that these RNA molecules adopt an extended solution structure. (**A**,**B**) Three structures representing sequential 90° rotational angles from the top panel structure. Dimensions represent the D_max_ obtained from P(r) analysis.

**Figure 5:**
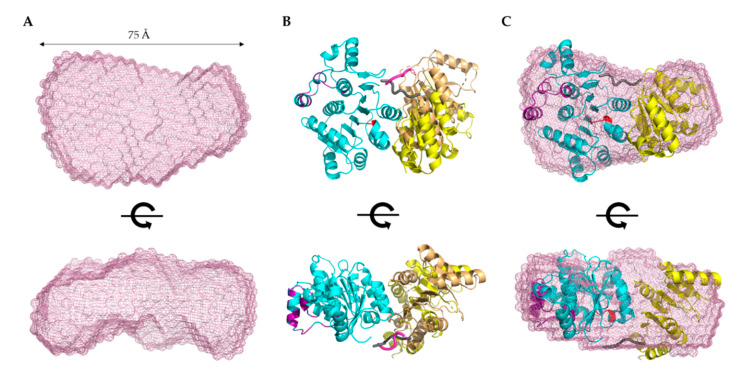
Structural modeling of DDX17_135–555_. (**A**) Low-resolution SAXS structure indicating that DDX17_135–555_ adopts an extended globular conformation in solution. The bottom panel represents a 90° rotation of the x-axis from the top panel. Dimensions represent the D_max_ from P(r) analysis. (**B**) CORAL-derived models of DDX17_135–555_, suggesting a linker (purple/gray chain) between the ATP-binding domain (blue ribbon), and the helicase domain (yellow/brown ribbon), allowing them to adopt different orientations. (**C**) SAXS envelope overlayed with the CORAL-derived representative model highlighting an agreement between high- and low-resolution models.

Next, we employed DAMMIN [26] to obtain low-resolution structures for each biomolecule, which involves a simulated annealing protocol allowing for the incorporation of P(r) data (D_max_ and R_g_ as constraints). Twelve models were calculated for all three biomolecules and all models have excellent agreement (X^2^) between the experimentally obtained scattering data and the calculated scattering data (Table 1). Following DAMMIN, we employed DAMAVER [27] for the alignment and rotation of all 12 models to gain an averaged filtered structure for each biomolecule, which represents averaged structural features from individual models (Figure 4 and Figure 5A) [27]. For each case, the overlap function, the normalized spatial discrepancy (NSD), was estimated to provide a measure of the goodness of fit of the superimposition of each model. Table 1 presents the NSD values for the 12 models calculated for each biomolecule, and the low values suggest that the models in each case are highly similar to each other. The models presented in Figure 4 and Figure 5A are the averaged filtered structures for NCR, 5’ IGR, and DDX17_135–555_, which indicate that both ncRNAs adopt extended structures in solution, while DDX17_135–555_ has a nearly globular conformation.

Recently, a high-resolution structure of DDX17 containing the ATP-binding and helicase domains (6UV0) was determined using X-ray crystallography [29]. We noticed a flexible linker between the ATP-binding and helicase domains, which could not be resolved in the high-resolution crystal structure. Therefore, we sought to use the scattering data of DDX17_135–555_ to perform high-resolution modeling using the program CORAL, as described elsewhere [30]. Using the crystal structure’s high-resolution information of the ATP-binding domain (155aa-382) and the helicase domain (389aa-555), we calculated 12 separate models and assessed their quality by comparing model-derived SAXS data with experimentally collected SAXS data. Each of the 12 models we calculated has X^2^ values of ~1.2, suggesting they are a good fit for the original data. This led us to believe that the helicase domain can adopt multiple different orientations in solutions, consistent with our initial low-resolution SAXS structure presented in Figure 5A. Figure 5B presents the CORAL-derived representative models, which highlight the relative orientations of the helicase domain due to the presence of a linker. Figure 5C demonstrates the overlay of the CORAL-derived model with the DDX17_135–555_ low-resolution structure, indicating an overall agreement between both approaches.

### 2.3. DDX17 Binds to the IGR and 5’NCR Non-Coding RNAs

**Figure 6:**
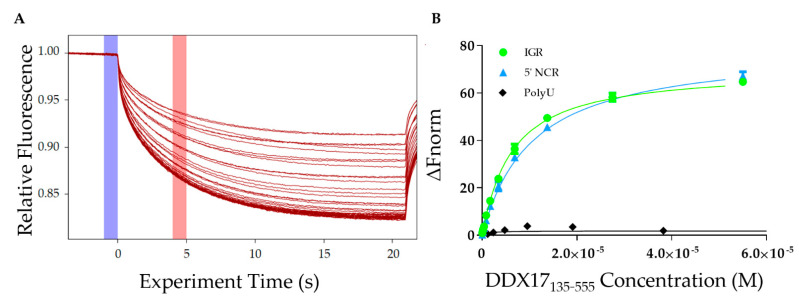
Interaction studies of DDX17_135–555_ with IGR and 5’ NCR. (**A**) Microscale thermophoresis (MST) traces indicating the change in fluorescence when exposed to the infrared laser. Each trace represents a different concentration of DDX17_135–555_ and is used to assess how the change in concentration affects the fluorescently labeled RNA migration. The blue highlight is the “cold” region and the red highlight is the “hot” region. The difference between these regions is used to calculate the ΔF_norm_. (**B**) The MST binding curves for the IGR and 5’ NCR RVFV RNAs (n = 3). RNA was used at a concentration of 40 nM while DDX17_135–555_ was titrated up to a maximum concentration of 55 µM. The y-axis ΔF_norm_ is the change in fluorescent migration normalized to 0. The dissociation constant for DDX17_135–555_ and the IGR was determined to be 5.78 ± 0.15 µM (reduced Χ^2^ = 0.967, Std. error of regression = 0.702) while for DDX17_135–555_ and the 5’ NCR was determined to be 9.85 ± 0.11 µM (reduced Χ^2^ = 0.996, Std. error of regression = 0.351). We used polyU RNA as a negative control (black diamonds) that did not bind to DDX17_135–555_.

After analyzing the homogeneity of DDX17_135–555_, RVFV S-segment IGR, and 5′ NCR, we determined the affinity of DDX17 for both ncRNAs using microscale thermophoresis (MST). MST is a powerful technique that allows for rapid interaction analysis by measuring the change in fluorescent migration as the molecules are excited via infrared laser [31,32]. DDX17_135–555_ was titrated against the fluorescently labeled RVFV RNAs. The addition of DDX17_135–555_ (the ligand) to the fluorescent RNA molecules (the target) causes them to migrate at a rate different than when DDX17_135–555_ is absent. A dissociation constant is determined by relating the change in fluorescent migration of the target to the concentration of the added ligand [33]. Figure 6A represents MST traces, where the blue highlight represents the “cold” region which is used to normalize the change of fluorescence measured in red, representing the “hot” region. Our MST studies demonstrate that DDX17_135–555_ interacts with IGR and 5’ NCR with dissociation constants of 5.77 ± 0.15 µM and 9.85 ± 0.11 µM, respectively (Figure 6B).

### 2.4. DDX17 Unwinds RVFV RNA in an ATP-Dependent Fashion

**Figure 7:**
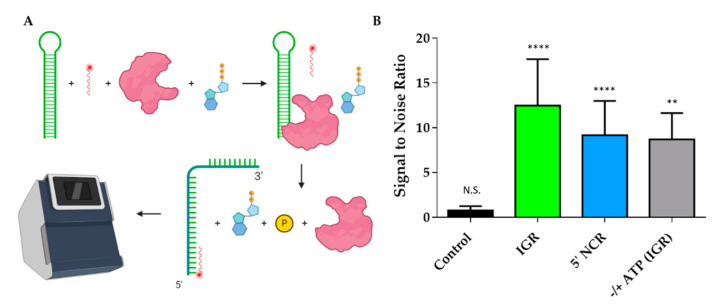
DDX17_135–555_ helicase assays performed using MST. (**A**) Representation of helicase assay using MST. The helicase assay was performed by combining the RNAs, fluorescently labeled DNA oligo, DDX17_135–555_, and ATP. DDX17_135–555_ hydrolyzes the ATP and unwinds the RNA, giving the oligo access to the newly opened complimentary site. The fluorescently labeled DNA oligo hybridized to the RNA can be measured in the MST by detecting the change in migration. This is compared to a control that uses bovine serum albumin (BSA) instead of DDX17_135–555_, and a change in the migration indicates that the RNA has been unwound by DDX17. (**B**) Signal to noise ratios of different comparative assays (n = 3). Control compared a reaction mix without protein to BSA, signal to noise did not meet the threshold of 5 and was not significant, unpaired *t*-test (*p* = 0.9350, N.S.). DDX17_135–555_ caused a significant change in the IGR (*p* < 0.0001, ****), with the signal to noise ratio reaching 12.5. The 5’ NCR also experienced a significant shift in the presence of DDX17_135–555_ (*p* < 0.0001, ****), having a signal to noise ratio of 9.17. Gray bar represents including ATP vs. not including ATP in the reaction mixture, showing ATP causes a significant change in fluorescent migration, having a signal to noise of 8.70 (*p* = 0.0059, **).

Since we confirmed that DDX17_135–555_ binds to both ncRNAs, we wanted to evaluate DDX17’s ability to unwind the RNAs. Figure 7A is a schematic representation of the experimental design which describes the overall approach of utilizing MST to perform a helicase assay. The signal to noise ratio, which is a measure of significance that uses the response amplitude of the MST traces, is indicated in Figure 7B. To assess statistical significance, we used unpaired *t*-tests. We determined that the fluorescent migration did not experience a significant change (*p* = 0.9350, signal to noise = 0.750) in the presence of bovine serum albumin (BSA) with the reaction mixture (RNA + fluorescent oligo + ATP), suggesting that BSA cannot unwind RNA, which makes it a suitable control for the subsequent experiments. Next, we compared the BSA reaction mixture to the reaction mixture with DDX17_135–555_. The results suggest that the addition of DDX17 with either IGR or 5’ NCR causes a significant change in the migration of fluorescence (*p* < 0.0001 for both, signal to noise = 12.5 and 9.17, respectively), indicating a binding event occurred upon the addition of DDX17. Collectively, our analysis demonstrates that DDX17_135–555_ can unwind the RNA, allowing the hybridization of the DNA oligo to the RNA(s). To determine the effect of ATP on the helicase activity of DDX17, we compared the reaction mix with and without ATP (gray bar). We observed that the presence of ATP resulted in a significant difference in fluorescence migration compared to without ATP (*p* = 0.0059, signal to noise = 8.70).

## 3. Discussion

The study performed by Moy et al. in 2014 [11] concluded, in vivo, that U2OS human cells infected with RVFV cause activation of DDX17 to restrict RVFV replication through an interferon-independent pathway. CLIP-seq analysis determined that DDX17 binds to two essential stem-loop regions on the RVFV S-segment RNA: IGR and 5′ NCR [11]. We, therefore, sought to characterize this interaction in vitro to substantiate that DDX17 is an interacting partner of RVFV ncRNAs.

We expressed and purified a construct that contains both the ATP-binding domain and the helicase domain, DDX17_135–555_ (Figure 1). Next, we transcribed, purified, and characterized the RVFV IGR and 5’ NCR ncRNAs in vitro. As AUC is a reliable and widely accepted technique to assess the solution state of biomolecules [34,35,36], we performed the SV-AUC experiments. The SV-AUC data suggested that both ncRNAs are relatively pure, with the presence of dimer and tetrameric assemblies (Figure 2B), which is similar to our prior study on Murrey Valley and Powassan virus ncRNAs where we also observed the presence of oligomeric species [20]. Similarly, SV-AUC studies also indicated that DDX17_135–555_ is mainly monomeric at the examined concentration. We also obtained the diffusion coefficients and the Stokes radii for DDX17_135–555_, IGR, and 5’ NCR (Table 1).

SAXS excels at being a complementary structural biophysical method by enabling solution structure studies of virtually all biomolecules, and their biomolecular complexes [18,22,30,37,38,39,40]. While SAXS structures are low resolution in comparison to high-resolution structures determined using X-ray crystallography or NMR, oftentimes obtaining high-quality crystals for crystallography or biomolecular labeling for NMR is challenging [18,38,40,41,42,43]. By employing HPLC-SAXS for data collection instead of traditional SAXS, we ensure that our collected scattering data will be monodispersed. These monodispersed preparations were confirmed by the linearity of fit in the low-q region using the Guinier analysis (Figure 3B). Using Guinier analysis, we also calculated R_g_ values for all three biomolecules (based on low-q region) (Figure 3B) and compared them to those calculated through P(r) analysis (Figure 3D). The R_g_ values for both analyses were highly similar (Table 1), which confirms that our data are reliable and it is worth proceeding with more analysis. Dimensionless Kratky analysis suggested that the IGR and 5’ NCR adopt an elongated structure (Figure 3C). Finally, the P(r) distribution (Figure 3D) reveals that both ncRNAs quickly increase to the maxima, and then steadily decrease, which suggests an elongated structure, as observed earlier [20,38]. Comparatively, the P(r) distribution of DDX17_135–555_ displays a skewed Gaussian distribution, suggesting that it adopts a more compact conformation compared to the ncRNAs (Figure 3D). We observe that both ncRNAs have different D_max_ (110 vs. 145 Å), despite having a similar length. The 5’ NCR, based on its D_max_, likely contains extended amounts of single-stranded regions (Figure 4A), whereas IGR could have a higher content of double-stranded structures (Figure 4B). IGR and 5’ NCR (Figure 4A,B) confirm that both RNAs adopt an elongated structure, as indicated by initial dimensionless Kratky analysis. The ratio of R_g_ to R_h_ is a good indicator of the solution conformation of biomolecules. Compact spherical biomolecules typically have an R_g_/R_h_ ratio of ~0.70. This ratio increases as the shape of the molecule changes from globular to extended conformation [44,45]. For IGR and 5’ NCR, the R_g_/R_h_ values are 1.35 and 1.43, suggesting that both ncRNAs have extended conformations. For DDX17_135–555_, we obtained an R_g_/R_h_ of 0.62, indicating that it is more globular than the ncRNAs.

The low-resolution structural modeling of DDX17 confirmed its extended globular nature (Figure 4A). A secondary strength of SAXS is the ability to combine high-resolution structures or homology models of individual domains, or computational studies with low-resolution SAXS models [18,30,38,41,42]. The crystal structure of DDX17 containing the ATP-binding and helicase domain (6UV0) was determined [29], allowing us to compare their high-resolution data to our low-resolution models to evaluate the validity of our models. Since the flexible linker between the ATP-binding and helicase domains was not resolved, we performed structural modeling using CORAL, which suggested that relative to the ATPase domain, the helicase domain exhibits conformational flexibility in solution (Figure 5C).

To establish the direct interaction between DDX17_135–555_ and both IGR and 5’ NCR ncRNAs, we performed MST assays as described previously [30,46,47]. Our analysis indicated that both RNAs interact with DDX17_135–555_. However, despite having relatively similar nucleotide length, IGR binds with a comparatively higher affinity to 5’ NCR (5.77 µM for the IGR vs. 9.85 µM for the 5’ NCR) (Figure 6B). Compared to the observations made for DDX5 (a DDX17 homolog), our results suggest that the DDX17 interacts with RVFV RNAs weakly (in µM range) [48,49]. However, an important distinction between previous studies and our work is that we have used considerably longer ncRNAs, and the minimalistic DDX17 construct. This could result in differences in specificity, nonetheless, we have demonstrated that our construct is specific to the RVFV RNAs and binds with them with different affinities. Considering how compact the IGR is, based on scattering analysis, it may indicate that DDX17 has tighter binding to double-stranded RNA regions. Although DDX17 is primarily located in the nucleus, its presence in the cytoplasm and ability to interact with RNAs, including the RVFV ncRNAs, suggests that DDX17 may act as a sensor for these viral RNAs within the cytoplasm [11,50], similar to other helicases and host proteins, like DDX3X and Protein Kinase R (PKR) [51,52]. Since DDX17 is a known helicase, we wanted to perform helicase assays to determine if DDX17_135–555_ can unwind RVFV ncRNA. Helicase assays are often conducted by using radioactivity or fluorescent resonance energy transfer (FRET)based analysis [53,54]. However, our endeavor to develop a time and cost-effective alternative led us to design a unique experiment using MST. MST is ideal for our experiment because of its sensitivity for binding events, the low concentrations of samples required, and the availability of the reaction components, other than fluorescently labeled DNA oligos [55,56]. Using this simple assay, we demonstrated that DDX17_135–555_ was able to unwind both RNAs and in a manner that is ATP dependent (Figure 7B). Currently, it is speculated that the ATP-binding domain hydrolyzes ATP to drive the helicase activity [57] which is consistent with our results. In conclusion, we have demonstrated that DDX17_135–555_ is capable of directly binding and unwinding the non-coding regions of the S-segment genome of Rift Valley fever virus. This suggests that it could be critical for recognizing non-coding regions from other viral RNA.

## 4. Materials and Methods

### 4.1. Protein Expression and Purification of DDX17_135–555_

The DDX17_135–555_ cDNA construct in the pOPINF vector was designed with the help from the Oxford Protein Production Facility (OPPF, Harwell Oxford, Didcot, UK). DDX17_135–555_ was expressed using Lemo21(DE3) *E. coli* cells. The culture was grown in Luria broth containing kanamycin (50 mg/mL) and chloramphenicol (100 mg/mL) antibiotics. The culture was then transferred to Terrific broth containing 5% glycerol, and the cells were grown at 37 °C in an orbital shaker for 5 h, followed by a reduction in temperature to 20 °C for 16–18 h, harvested by centrifugation, and resuspended in lysis buffer (50 mM Tris, 500 mM NaCl, 10 mM imidazole 3 mM 2-Mercaptoethanol, 10mg/mL Lysozyme, 0.1% Tween-20, and 5% glycerol). The resulting cell suspension was sonicated and centrifuged at 30,000× *g*. The supernatant was filtered through a 0.45 µm syringe filter to prepare for chromatography.

Nickel affinity purification was performed using the ÄKTA start protein purification system (Global Life Science Solutions USA LLC, Marlborough, MA) with the HisTrap™ High-Performance column (Global Life Science Solutions USA LLC, Marlborough, MA) via the hexahistidine tag on DDX17_135–555_. Protein was eluted in 2mL fractions using an imidazole gradient up to 500 mM. Further purification and buffer exchange were performed using an ÄKTA pure purification system (Global Life Science Solutions USA LLC, Marlborough, MA) using Superdex^®^ 200 10/300 GL (Global Life Science Solutions USA LLC, Marlborough, MA). DDX17_135–555_ was eluted in 50 mM Tris, 150 mM NaCl, and 3% glycerol. Elutions containing DDX17_135–555_ were pooled and concentrated using Amicon^®^ Ultra-15 Centrifugal Filter Units (30,000 MWCO, Millipore Canada Ltd, Etobicoke, ON). The 110 µM DDX17_135–555_ stocks were aliquoted and frozen in liquid nitrogen before being stored at −80 °C. 

### 4.2. Preparation of Rift Valley Fever Virus Non-Coding RNAs

The cDNA sequences were prepared under T7 RNA polymerase control, with two additional G nucleotides on the 5′ end followed by an XbaI restriction enzyme cut site (T^CTAGA) on the 3’ end. Both RVRV constructs were designed based on the Genebank sequence of EU312119.1. The underlined regions are the complimentary regions to our fluorescent oligos described in a later section. Both RNA constructs used in the experiments are listed as follows:

1. RVFV NCR S Segment 812–886

5′GGAUUUGUUGAGGUUGAUUAGAGGUUAAGGCUGCCCCACCCCCCACCCCCUAAUCCCGACCGUAACCCCAACUCCU3’

2. RVFV IGR S Segment 25–100

5′GGCAAGUAUAUCAUGGAUUACUUUCCUGUGAUAUCUGUUGAUUUGCAGAGUGGUCGUCGUGUUGUGUCAGUGGAGUACAU3′

Each RNA was prepared using an in vitro transcription reaction using T7 RNA polymerase (made in-house) followed by purification using a Superdex^®^ 200 10/300 GL via an ÄKTA pure system (Global Life Science Solutions USA LLC, Marlborough, MA). Fractions were analyzed using urea-polyacrylamide gel electrophoresis (urea-PAGE): 10 µL of each fraction were mixed with 2 µL of RNA loading dye and loaded into a 1.0 cm well PAGE (Bio-Rad Laboratories (Mississauga, ON). The urea-PAGE (7.5%) was then developed at 300 V, room temperature for 25 min in 0.5× TBE, followed by staining and visualization with Sybr Safe (Thermofisher Scientific, Saint-Laurant, QC, Canada). Fractions containing a single band were used for further experimentation. Fractions containing the purified RNA of interest were concentrated by ethanol precipitation, and each pellet was resuspended in RNA buffer (10 mM Tris pH 7.5, 100 mM NaCl, and 5 mM MgCl_2_).

### 4.3. Fluorescent Labeling of RNA

RNAs were incubated on ice for 30 min in 0.1M sodium acetate (pH 5.3) and 2 mM potassium periodate. Following incubation, the reaction was stopped by adding ethylene glycol to a concentration of 10 mM and incubated again on ice for 10 min. We then performed two ethanol precipitations, resuspended the RNA in water, along with 0.1 M NaOAc and 10 mM fluorescein-5-thiosemicarbazide (FITC), and incubated the mixture on ice and in the dark for 16 h. Following incubation with the fluorescent dye, the mixture was phenol extracted (1 vol phenol:1 vol mixture) 5 times until the phenol layer no longer changed color, indicating all free dye had been removed from the RNA mixture. We then ethanol precipitated the resulting labeled RNA twice, followed by resuspension in RNA buffer.

### 4.4. Analytical Ultracentrifugation (AUC)

We collected SV-AUC data for FPLC-purified RNA and protein using a Beckman Optima AUC centrifuge with an AN60-Ti rotor at 20 °C. Each sample was loaded into Epon-2 channel centerpieces and was measured at 0.5 OD_260_ for RNA (680 nM) and 0.5 OD_280_ for protein (10.2 µM). For SV-AUC experiments, we used 10 mM Tris and 500 mM NaCl with 5 mM mgCl_2_ buffer at pH 7.5 for RNA and 50 mM Tris, 150 mM NaCl, and 5% glycerol buffer at pH 8 for protein. Intensity scans were collected at 20 s intervals at 40,000 revolutions per minute, measuring at 20 °C. All data were analyzed using UltraScan-III [58] according to the workflow described elsewhere [59]. Finite element fits were processed on the Lonestar5 (Texas Advanced Computing Center, Austin, TX, USA) and Comet (San Diego Supercomputing Center, San Diego, CA, USA) supercomputers. The collected SV-AUC data were analyzed using two-dimensional spectrum analysis (2DSA) to subtract time and radially invariant noise components and to fit the meniscus and bottom positions [60], followed by genetic algorithm analysis combined with Monte Carlo analysis [61]. The buffer density and viscosity corrections were calculated with UltraScan (1.0030 g/cm^3^ and 1.0100 cP, respectively, for the RNA buffer and 1.017 g/cm^3^ and 1.152 cP for the protein buffer). Partial specific volumes of 0.55 mL/g [20] and 0.732 mL/g [62] were assumed for RNAs and protein, respectively. All reported hydrodynamic parameters are corrected to standard conditions (20 °C and water), as implemented in UltraScan [58].

### 4.5. Microscale Thermophoresis RNA and Protein Binding Studies

A two-fold serial dilution was performed on DDX17_135–555_ where the highest concentration was 55 µM (as presented in Figure 6B). A constant amount of FITC-labeled RVFV NCR, or 5’ IGR, was added to each serial dilution of DDX17_135–555_, resulting in a final concentration of 40 nM. The final concentration of polyU (negative control, Sigma-Aldrich Canada) in each assay was 50 µg/mL, and the initial fluorescence was similar to the ncRNA experiments. Samples were incubated together at room temperature for 10 min and then added to Nanotemper Technologies Monolith^®^ NT.115 instrument (Munich, Germany) hydrophobic capillaries and loaded onto the MST block. Thermophoresis was measured at an ambient room temperature of 25 °C and performed using 20% excitation power for RVFV NCR and 40% for 5′ IGR (blue filter) and medium MST IR-laser power. Fluorescent migration used to determine *K_d_* was measured from 4.0 to 5.0s and then normalized to initial fluorescence (−1.0 to 0s). The data from three independent replicates were analyzed using MO Affinity Analysis software v2.1.3 and fit to the standard *K_d_* fit model, which describes a molecular interaction with a 1:1 stoichiometry according to the law of mass action. *K_d_* is estimated by fitting Equation (1), where *F(c)* is the fraction bound at a given ligand concentration c; Unbound is the *F_norm_* signal of the target alone; *Bound* is the *F_norm_* signal of the complex; *K_d_* is the dissociation constant; and *c_target_* is the final concentration of the target in the assay.

(1) $$F\left(c\right)=Unbound+\left(Bound-Unbound\right)\;\;\;\;\;\;\;\;\;\;\;\;\;\;\;\;\;\;\times \frac{c+c_{target}+K_{d}-\sqrt{{\left(c+c_{target}+K_{d}\right)}^{2}}-4cc_{target}}{2c_{target}}$$

### 4.6. Helicase Assay

Firstly, we input our sequences into sfold [63] to determine the theoretical secondary structure and identified a portion of each RNA molecule that was double stranded. Oligos with complementary sequences to the double-stranded region(s) of the RNA(s) analyzed here were synthesized with a 5’ conjugated Cy5 fluorophore. The region of each RNA molecule which the oligos hybridize to is underlined, as described above (4.3). The sequences for RVFV 5’ IGR and RVFV 5’ NCR oligo(s) are: 5′Cy5/CAACTCCAACTAATCTCCA3’ and 5′Cy5/AGACAACTAAACGTCTCAC3’, respectively. 

Using Monolith^®^ NT.115 that assesses the change in fluorescence migration, we were able to determine if the RNA molecules were unwound, thus allowing the oligo to bind to the now exposed complementary RNA. The reaction mixture contains 40nM of Cy5-DNA oligos, 1 µM of the RNA, and 4.25 mM of ATP. To test the helicase activity of DDX17_135–555_, we added the enzyme to a final concentration of 20 µM. As a control, we compared the unwinding activity of bovine serum albumin (BSA) with the activity observed in the absence of any protein (black bar). For the BSA control, the same concentration was used as for DDX17 (green and blue bars). Additionally, to assess the importance of ATP in unwinding activity, we compared DDX17_135–555_ without ATP to DDX17 with ATP (gray bar). Each run uses 4 capillaries, and we performed 3 runs for each condition before using the MO Affinity Analysis software to analyze the data. The analysis software assesses the signal to noise ratio between a run with and without the protein. Signal to noise is a measure of the response amplitude that is divided by the noise of the environment, and Equation (2) represents how this can be calculated [64]. If the signal to noise ratio rises above 5, the assay indicates that a binding event has occurred. To further analyze the helicase assay, unpaired *t*-tests were performed

(2) $$S/N=\frac{ResponseAmplitude}{\sqrt{\frac{\sum_{i}{\left(r_{i}-\overset{´}{r}\right)}^{2}}{n-1}}}$$

### 4.7. Small-Angle X-ray Scattering

Small-angle X-ray scattering was performed by utilizing the B21 BioSAXS beamline at Diamond Light Source (Didcot, Oxfordshire, UK) to collect high-performance liquid chromatography SAXS (HPLC-SAXS) data which can be found described previously [65]. Using a specialized flow cell connected to an in-line Agilent 1200 (Agilent Technologies, Stockport, UK) HPLC, 50 µL of each purified sample (protein or RNA) were injected onto a Shodex KW403-4F (Showa Denko America Inc., New York, NY, USA) size exclusion column pre-equilibrated with buffer, at a flow rate of 0.160 mL per minute. X-rays were exposed to each frame for 3 s. The peak region for each sample was buffer subtracted using baseline measurements and merged using Primus [66] or ScAtter [67], as previously described. The merged data were analyzed initially by Guinier approximation [21] to obtain the radius of gyration (R_g_) and evaluate homogeneity. Dimensionless Kratky analysis [23] was performed on all samples to evaluate the folding extend of the biomolecules of interest, which is reviewed in detail elsewhere [22]. Following Kratky analysis, we performed a pair distance distribution (P(r)) analysis using GNOM [24] to additionally provide the R_g_ and the maximum particle dimension (Dmax). Using the information from the P(r) plot, we generated models using DAMMIN [26], without enforced symmetry, which can be found previously described [30]. Finally, the resulting models were averaged and filtered to generate a single representative averaged model using DAMAVER [27,43,68]. 

Recently, a crystal structure of DDX17 containing the ATP and helicase domain (6UV0) was published [29]. We used the scattering data of DDX17_135–555_ and performed high-resolution modeling, using the crystal structure and CORAL program, as described earlier [30]. Briefly, the high-resolution structure information of the ATP domain (155aa–382) and helicase domain (389aa–555) was provided as input data along with the raw scattering data, and the residues 383–388 were used as a flexible linker. Using this approach, we initially calculated 12 models and the quality of the models was assessed using Χ^2^ values.

## Figures and Tables

**Table 1:** Solution properties of DDX17_135–555_, IGR, and 5’ NCR.

Sample	DDX17_135_555_	IGR	5’ NCR
*M_w_* (kDa, sequence)	48.45	23.82	24.70
Sedimentation coefficient, S (10^−13^ s) ^∇^	3.16	4.07	4.18
Diffusion coefficient D (10^−7^ cm^2^/s) ^∇^	5.22	7.62	6.58
R_h_ (Å) ^∇^	41.06	28.11	32.64
I(0) ^#^	0.003 ± 2.5 × 10^−^^5^	0.026 ± 4.4 × 10^−^^5^	0.022 ± 2.6 × 10^−^^4^
q.Rg range ^#^	0.39–1.30	0.26–1.29	0.40–1.29
R_g_ (Å) ^#^	24.78 ± 0.36	36.42 ± 0.10	50.44 ± 0.88
I(0) ^∆^	0.003 ± 2.3 × 10^−^^5^	0.026 ± 4.3 × 10^−^^5^	0.019 ± 1.7 × 10^−^^4^
R_g_ (Å) ^∆^	25.46 ± 0.27	38.00 ± 0.08	46.66 ± 0.34
D_max_ (Å)^∆^	79.21	122	148
Χ^2^ *	~1.00	~1.10	~1.30
NSD *	0.52 ± 0.02	0.73 ± 0.02	0.58 ± 0.01

The *M_w_* values were calculated using nucleotide sequences. ^∇^—determined using SV-AUC analysis and UltraScan-III package [28]. Sedimentation coefficients obtained following genetic algorithm–Monte Carlo analysis. **^#^**—obtained from Guinier analysis [21]. **^∆^**—determined using *P*(*r*) analysis using the GNOM program [24]. *—values derived from DAMMIN [26] and DAMAVER [27] analysis. Rh – hydrodyhamic radius; Rg – radius of gyration; Dmax – maximum particle dimension; NSD: normalized spatial discrepancy.

## Data Availability

The data will be available from the corresponding author on reasonable request.

## Abbreviations

MST	Microscale Thermophoresis
AUC	Analytical Ultracentrifugation
ncRNA	Non-coding RNA
rRNA	Ribosomal RNA
IGR	Intergenic Region
SAXS	Small-angle X-ray Scattering
5′ NCR	5′ Non-coding RNA
SV	Sedimentation Velocity
BSA	Bovine Serum Albumin
RVFV	Rift Valley Fever Virus
FITC	Fluorescein Isothiocyanate
RNA	Ribonucleic Acid
FPLC	Fast Protein Liquid Chromatography
HPLC	High-performance liquid chromatography
PAGE	Polyacrylamide Gel Electrophoresis
SEC	Size-Exclusion Chromatography
